# Consumption of soya isoflavones improved polycystic ovary syndrome-associated metabolic disorders in a rat model

**DOI:** 10.1017/S0007114524001296

**Published:** 2024-08-28

**Authors:** Chao-Wu Xiao, Adriana A. Carbonel, Patricia D. A. Lima, Amy Hendry, Benjamin K. Tsang

**Affiliations:** 1 Nutrition Research Division, Food Directorate, Health Products and Food Branch, Health Canada, Banting Research Centre, Ottawa, ON K1A 0K9, Canada; 2 Food and Nutrition Science Program, Department of Chemistry, Carleton University, Ottawa, ON K1S 5B6, Canada; 3 Chronic Disease Program, Ottawa Hospital Research Institute, Ottawa, ON K1H 8L6, Canada; 4 Department of Morphology and Genetics, Paulista School of Medicine, Federal University of São Paulo, São Paulo, Brazil; 5 Queen’s CardioPulmonary Unit, Department of Medicine, Queen’s University, Kingston, ON, Canada; 6 Departments of Obstetrics & Gynecology and Cellular & Molecular Medicine, Interdisciplinary School of Health Sciences, University of Ottawa, Ottawa, ON K1H 8L6, Canada

**Keywords:** Soya isoflavones, Insulin resistance, Non-alcoholic fatty liver disease, Polycystic ovary syndrome, Rat

## Abstract

Polycystic ovary syndrome is associated with increased risks for certain metabolic disorders such as insulin resistance, non-alcoholic fatty liver disease and suppressed ovarian follicular development. This study aimed to examine whether soya isoflavones (ISF) mitigate these polycystic ovary syndrome-associated metabolic disorders in a rat model. Weanling Sprague-Dawley female rats were randomly divided into six groups and were treated with either 0 or 83 µg/d dihydrotestosterone (DHT) to induce polycystic ovary syndrome and fed diets containing 0, 0·5, or 1 g ISF/kg diet for 8 weeks. DHT treatment increased food intake, body weight gain (*P* < 0·001), percentage of primordial follicles (60 % *v*. 50·9 %, *P* < 0·05) and accumulation of lipid droplets in the livers. It also elevated serum total cholesterol, free cholesterol, TAG, NEFA and leptin and hepatic total cholesterol and NEFA. Additionally, DHT treatment reduced the percentage of primary follicles (13·8 % *v*. 30·2 %, *P* < 0·05), ovary weight and length (*P* < 0·001), as well as insulin sensitivity (*P* < 0·01) compared with the Control. ISF intake at 1 g/kg reduced body weight gain, serum total cholesterol, free cholesterol, NEFA, leptin and hepatic TAG and DHT-induced insulin resistance (*P* < 0·01). ISF intake at both levels decreased DHT-induced lipid droplet accumulation in the livers and changes in the percentages of primordial and primary follicles. Dietary soya ISF alleviated DHT-induced body weight gain, insulin resistance and hepatic lipid droplet accumulation, as well as suppressed ovarian follicular development. This suggests that the consumption of soya foods or ISF supplements may be beneficial for individuals with polycystic ovary syndrome, mitigating the associated metabolic disorders such as diabetes and non-alcoholic fatty liver disease.

Polycystic ovary syndrome (PCOS) is a multifactorial endocrine condition and a heterogeneous syndrome with complex pathologies such as follicle growth arrest at the small antral stage, minimal granulosa cell proliferation, hyperthecosis, hyperandrogenemia and chronic anovulation^(1)^. PCOS is the most common endocrine disorder, affects about 6–15 % of women at reproductive age^(2)^ and accounts for 75 % of anovulatory infertility^(3–5)^. Its incidence shows a tendency to increase in most countries around the world^(6)^. PCOS is also associated with an increased risk of metabolic disorders such as insulin resistance, diabetes, obesity, hypertension, dyslipidemia, fatty liver and CVD^(7–10)^. For instance, the prevalence of non-alcoholic fatty liver diseases (NAFLD) in patients with PCOS^(5)^ ranged from 34 to 70 % compared with 14 to 34 % in healthy women. Conversely, women with NAFLD are more often diagnosed with PCOS^(2,11)^. Indeed, about 50 % of women with PCOS are those who have obesity, and particularly, abdominal obesity is common in these women^(12)^.

Obesity and insulin resistance are the major pathophysiological factors leading to the development of NAFLD in PCOS^(8)^. PCOS has been redefined as a reproductive and metabolic disorder because of the important role of insulin resistance in the pathophysiology of the syndrome^(13)^. However, the mechanism(s) involved in the development of metabolic disorders associated with PCOS is not fully understood, and whether hyperandrogenemia plays a role in this process remains to be determined. Lifestyle modifications including diet, weight loss and exercise are the most appropriate and the main strategy as therapeutic interventions for patients with PCOS and NAFLD^(13)^.

Isoflavones (ISF) are the major soya phytoestrogens, including genistin, daidzin and glycitein. Both genistin and daidzin are present as glycosides in soyabeans and can be hydrolysed and converted to aglycones, genistein and daidzein by intestinal microflora before they can be absorbed in the body. Soya ISF are structurally similar to endogenous oestrogen, and particularly genistein and daidzein can bind to both oestrogen receptors (ER) *α* and β with greater affinity to ERβ. Additionally, daidzin and daidzein can be metabolised to equol by intestinal bacteria in about 25–30 % of adults of Western countries and 50–60 % of adults in Japan, Korea or China or in Western adult vegetarians. Most of animal species, particularly rodents, can efficiently convert daidzin/daidzein to equol^(14)^. Equol has a much higher ER binding affinity than its precursor daidzein^(15)^. Soya ISF or genistein has been shown to improve PCOS pathophysiological factors and associated metabolic disorders in patients with PCOS. For example, genistein improved total cholesterol (TC) levels and reduced LDL-cholesterol, the LDL:HDL ratio^(16)^, TAG, dehydroepiandrosterone sulfate and testosterone^(17)^. Soya ISF improved insulin resistance and reduced the free androgen index, TAG and oxidative stress^(18)^. It is believed that consumption of soya ISF or genistein may prevent cardiovascular and metabolic disorders in patients with PCOS by improving their reproductive hormonal and lipid profiles^(17)^.

5*α*-Dihydrotestosterone (DHT) is a metabolite of testosterone and nonaromatizable androgen^(19)^. Treatment with DHT in female rats increased body weight gain (BWG) and insulin resistance^(20)^, similar to the observations in the patients with PCOS. However, whether DHT treatment results in dyslipidemia or liver lipid accumulation and whether consumption of soya ISF prevents or mitigates the effects of DHT remain to be determined. Using the DHT-induced rat model of PCOS, this study aimed to examine (a) whether DHT causes dysregulation in lipid metabolism and increases lipid accumulation in the liver, resulting in NAFLD, and (b) if dietary supplementation with soya ISF prevents or mitigates the effects of DHT in lipid metabolism, insulin resistance and ovary histomorphology.

## Materials and methods

### Animals, diets and DHT-induced PCOS

The animal experimental protocol (no. OHRI-1624-R1 A1) was approved by the University of Ottawa Animal Care and Usage Committee, and all animal procedures were performed in accordance with the Guidelines for the Care and Use of Laboratory Animals of the Canadian Council on Animal Care. The reporting in this paper followed the recommendations in the Animal Research Reporting of In Vivo Experiments guidelines^(21)^. Weanling Sprague-Dawley female rats at the age of 21 d were purchased from Charles Rivers (St. Constant, Quebec, Canada) and housed individually on a 12:12 h light:dark cycle, with free access to food and water. The studies were conducted in four cohorts. The rats were randomly divided into six groups, with eight rats per group for cohort 1 and six rats per group for the other three cohorts using a stratified randomisation method based on the body weights. The sample size calculation was conducted based on blood TC concentrations with a 25 % sd to detect a 15 % reduction. The power of the experiment was set to 80 %. A minimum sample size of twenty-six was considered necessary. The study is a two-way randomisation trial with 156 rats. Most analyses reported in this paper were conducted in the animals of cohort 1, with the exception of measuring serum lipid concentrations in all four cohorts and liver lipid content in cohorts 2–4 because the livers of the rats in cohort 1 were collected for the analysis of histology, protein and gene expression.

After acclimation of 1 week on a 20 % casein diet, the grouped rats were randomly assigned to receive a subcutaneous implant with either an empty (0 µg/d, Sham Control) or a DHT-filled silicone capsule (SILASTIC brand) that continuously releases 83 µg/d of DHT to induce PCOS^(20,22)^. The implanted rats were fed for 8 weeks with diets containing either 0, 0·5 or 1 g/kg diet of soya ISF from NovaSoya (Archer Daniels Midland), an alcohol extract from the preparation of soya protein isolate, containing 30 % total ISF, with a ratio of genistin:daidzin:glycitein = 1:1·3:0·3, and 70 % other compounds including 13 % saponins, 26 % other natural soya phytocomponents, 9 % protein, 11 % sugars, 4 % dietary fibre, 1 % fat and 6 % moisture (online Supplementary Fig. 1). The rats in cages were randomly located on the rack. All diets were formulated according to the specifications for the AIN93G diet, and the addition of NovaSoya was at the expense of maize starch and balanced by ISF-depleted alcohol extract of soya protein. All diets were isoenergetic and isonitrogenous, and food intake and body weight were recorded weekly. After the overnight fast, all animals were killed for the collection of blood, liver and ovaries. Ovary weight and length were measured in the rats of cohort 1, and the ovaries and a portion of liver tissue from the same area of the lobe in the rats of cohort 1 were fixed in buffered 4 % paraformaldehyde at 4°C for 24 h and then embedded in paraffin for histological assessment. Blood was kept overnight at 4°C to allow clotting and then centrifuged for serum separation.

### Assessment of oestrous cycle regularity

Vaginal lavages were performed daily at 9.00 hours using PBS (Sigma) for 2 weeks prior to the end of the experiment in the rats of cohort 1. Vaginal lavages were placed on slides, dried and stained with Giemsa. The regularity of the oestrous cycle was scored under light microscopy based on the rodent cyclicity criterion^(23)^. The existence of cell types, such as infiltrated leukocytes and nucleated and cornified epithelial cells, were assessed for the identification of the phases of the oestrous cycle^(24,25)^. The vaginal smears were classified into one of the four stages of the oestrous cycle, as described^(26)^.

### Insulin sensitivity test

The insulin sensitivity test was conducted 1 week prior to the completion of the experiment on rats in cohort 1 after overnight fasting. Human insulin at a dosage of 0·2 U/100 g body weight (Novo Nordisk Canada Inc.) was administered intravenously to all groups of the rats (*n* 8 rats/group) via the tail vein. Blood samples were collected from the saphenous vein at 0, 5, 10, 20, 40, 80, 160 and 320 min after insulin injection. Plasma glucose levels were determined using glucose test strips (Accu-Chek, Roche). The insulin sensitivity index K_ITT_ (rate constant for insulin tolerance test) was calculated as K_ITT_ = (0·693/t_1/2_) × 100, where t_1/2_ represents the half-life of glucose decay after insulin injection. A lower K_ITT_ indicated decreased insulin sensitivity or increased insulin resistance^(27)^.

### Histological analysis of ovaries and livers

After euthanasia, the ovaries and livers of three rats per group, randomly selected from cohort 1, were collected. Ovaries were weighed, while ovaries and livers were fixed in buffered 4 % paraformaldehyde at 4°C for 24 h before being embedded in paraffin. Sections of 5 μm thickness were stained with haematoxylin and eosin. Additionally, a portion of the livers from the same lobe of each rat was collected and embedded in Tissue-Tek O.C.T compound (Electron Microscopy Sciences, PA, USA) and immediately frozen.

### Oil red O staining and quantification of lipid droplets in the livers

Cryosections (7 µm) of liver tissues of the rats in cohort 1 were fixed in buffered 4 % paraformaldehyde at room temperature for 10 min and stained in oil red O solution (Electron Microscopy Sciences) as manufacturer’s instructions, counterstained with haematoxylin and mounted using an aqueous mounting medium (Vecta Shield). The images were taken with a 20× objective using the Zeiss Axioplan microscope (Zeiss, North York, Canada), and the Axion Vision software (Axion Vision software, Zeiss). The analysis was conducted using ImageJ (National Institutes of Health).

### Measurement of hepatic and serum lipids

Total lipids were extracted from the whole liver tissues using the chloroform-methanol method^(28)^. TC, TAG and NEFA in liver extracts, as well as TC, free cholesterol (FC), HDL and LDL-cholesterol and TAG in serum samples of the rats in all four cohorts, were measured using Wako assay kits (Wako Chemicals USA, Inc., Richmond, VA, USA) and the 96-well microplate analysis method to minimise variations. Serum leptin concentrations were determined using a rat leptin ELISA kit (Crystal Chem USA).

### Statistical analysis

Results are expressed as mean (sem), unless otherwise specified. All data were assessed for equality of variance prior to statistical analysis. Variables with skewed distribution were logarithmically transformed. Two-way ANOVA was used to examine the influence of ISF on PCOS by assessing the effects of DHT and soya ISF as well as their interactions. Differences between individual group means were determined by the Bonferroni *post hoc* test. The follicle scores in the ovaries were analysed using the *χ* ^*2*^ test. A probability of *P* < 0·05 was considered to be significant. All data were analysed using GraphPad Prism 5·0 Statistical software (GraphPad).

## Results

### Food intake, body weight gain, ovary weight and length

**Fig. 1.**
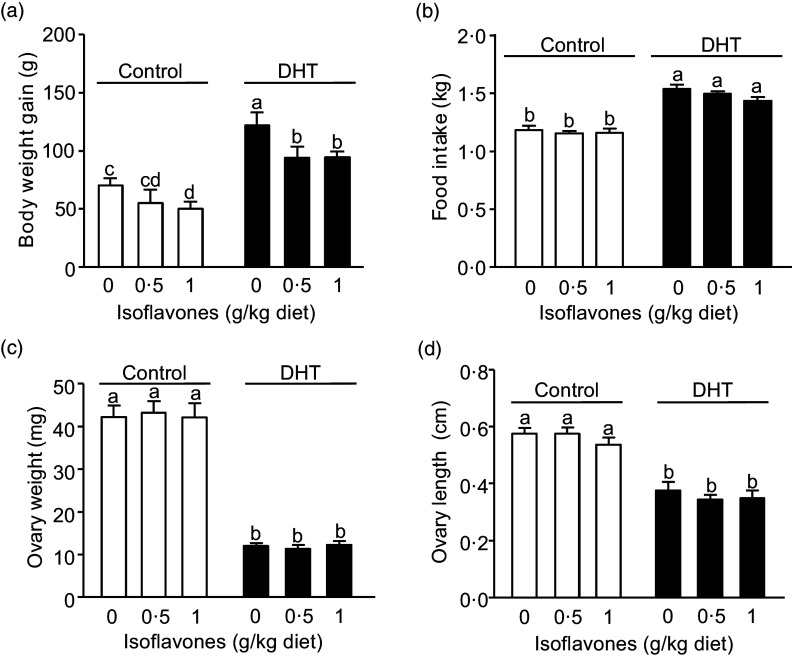
Body weight gain (a), food intake (b), ovary weight (c) and length (d) of the rats treated with 0 µg/d (Control, open bars) or 83 µg/d (DHT, solid bars) of dihydrotestosterone and fed diets containing 0, 0·5, or 1 g/kg diet of soya isoflavones for 8 weeks. Values are mean (sem), *n* 8. Means with different letters within Control or DHT groups differ, *P* < 0·05.

The rats fed 1 g/kg diet of soya ISF but not treated with DHT had lower BWG than the Control (< 0·05, Fig. 1(a)). PCOS group (DHT-treated) exhibited a significantly higher food intake (*P* < 0·01, Fig. 1(b)) and BWG compared with the non-PCOS (Sham Control) (*P* < 0·01), and both levels of soya ISF attenuated the BWG increase in PCOS group (*P* < 0·05, Fig. 1(b)). DHT treatment markedly reduced both ovary weight and length compared with the Control (*P* < 0·01, Fig. 1(c) and (d)).

### Blood glucose levels and insulin sensitivity

**Fig. 2.**
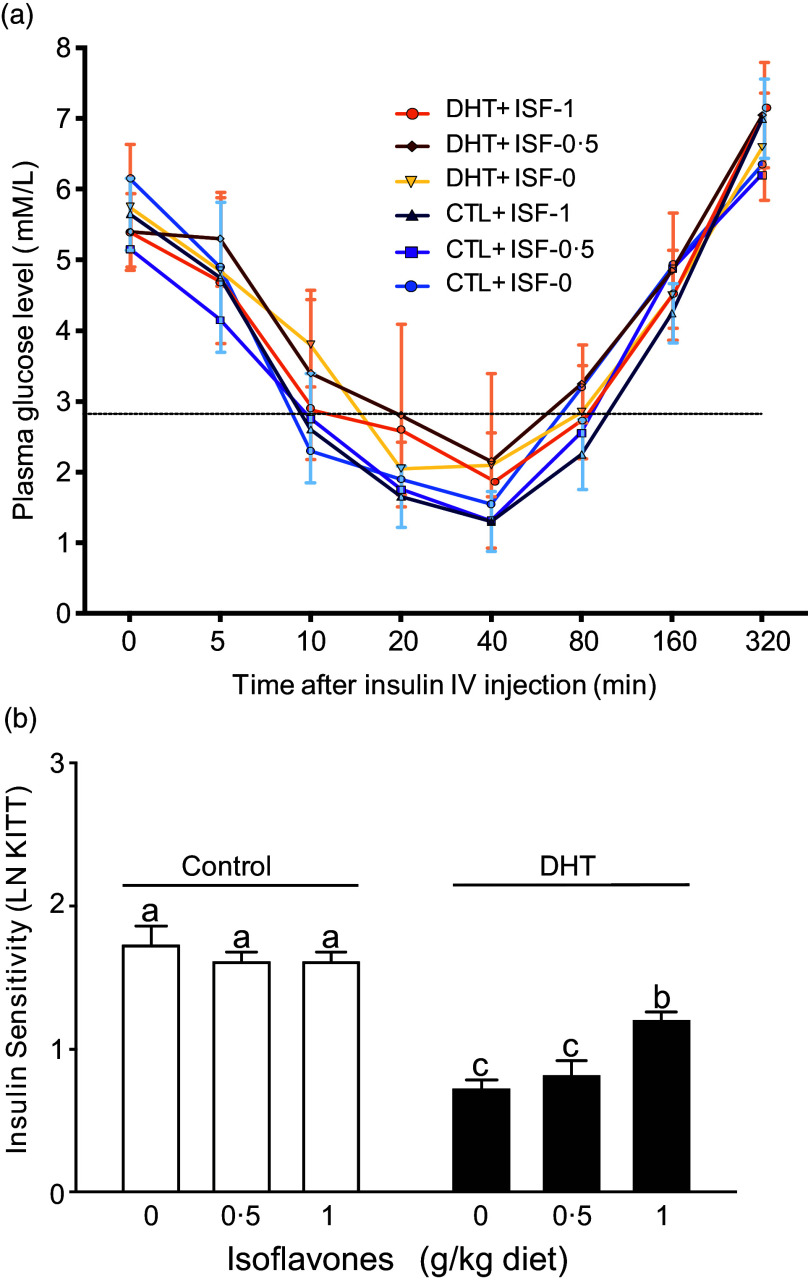
Plasma glucose levels (a) and insulin sensitivity (b) of the rats treated with 0 µg/d (Control, open bars) or 83 µg/d (DHT, solid bars) of dihydrotestosterone and fed diets containing either 0, 0·5, or 1 g/kg diet of soya isoflavones for 8 weeks. Values are mean (sem), *n* 8. Means with different letters within Control or DHT groups differ, *P* < 0·05.

The rats treated with DHT (PCOS rats) had higher plasma glucose levels (*P* < 0·001, Fig. 2(a)) and lower insulin sensitivity as measured by K_ITT_ (*P* < 0·001, Fig. 2(b)) after injection with insulin compared with the Control groups. Consumption of soya ISF at a level of 1 g/kg diet increased the insulin sensitivity in PCOS rats compared with 0 or 0·5 g/kg ISF diet (*P* < 0·01, Fig. 2(b)).

### Hepatic histology and accumulation of lipid droplets

**Fig. 3.**
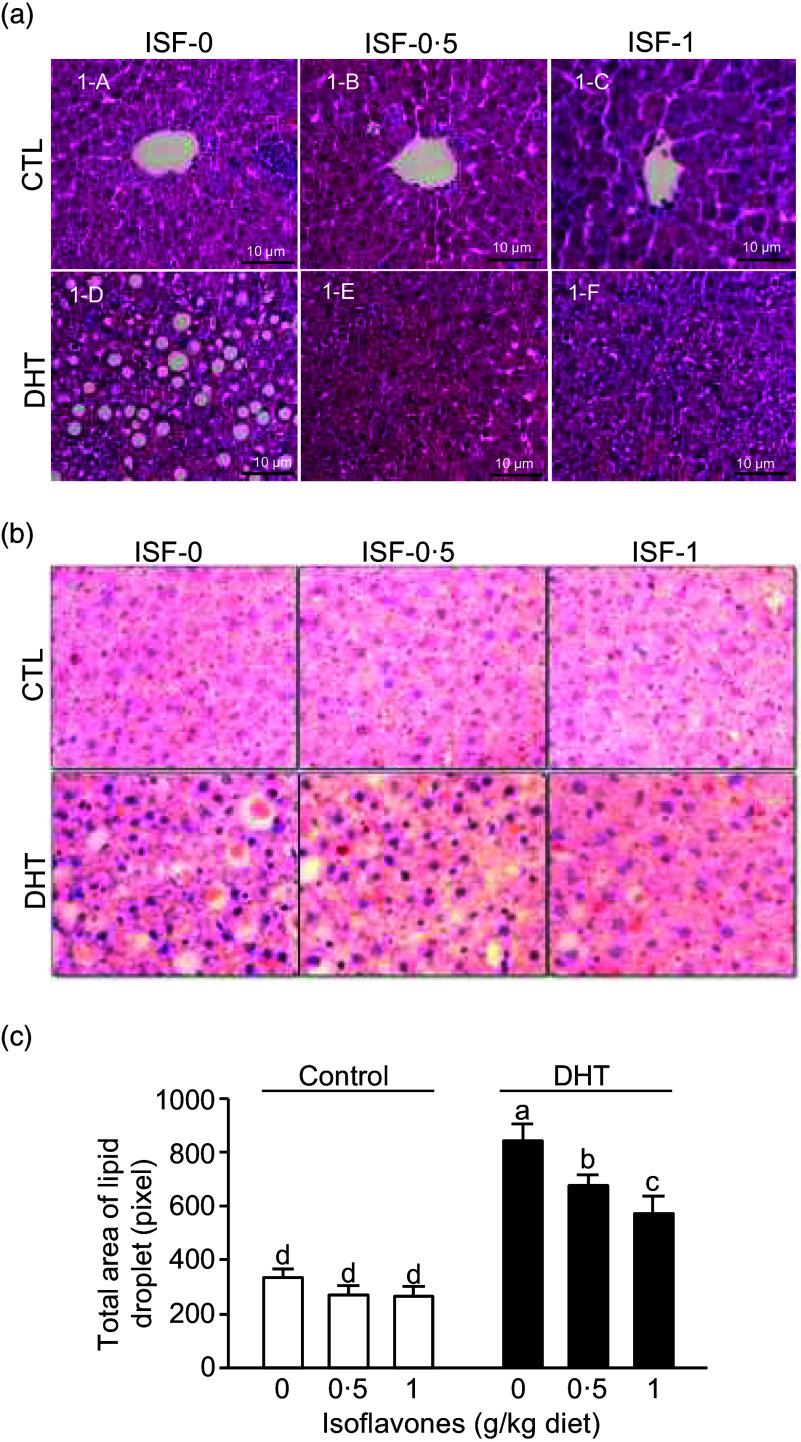
Histomorphology of the liver after stained with haematoxylin and eosin (a) or oil red O staining (b) in the rats treated with 0 µg/d (Control) or 83 µg/d dihydrotestosterone (DHT) and fed diets containing either 0, 0·5 or 1 g/kg diet of soya isoflavones (ISF) for 8 weeks. The total areas of lipid droplets in the liver sections were measured (c). The images shown are representatives of ten replicates of each treatment group. Values are mean (sem), *n* 3. Means with different letters within Control or DHT groups differ, *P* < 0·05.

The livers of all rats without DHT treatment showed a normal histological structure regardless of their ISF intake (Fig. 3(a)-A, -B and -C). In the PCOS rats not fed with any ISF (Fig. 3(a)-D), the hepatocytes in the hepatic parenchyma contain focal or generalised vacuoles with a micro or vesicular macro aspect, associated with the presence of sinusoid dilatation and progressive loss of the general structure of the tissue, which is consistent with steatosis. A moderate intensity of mononuclear inflammatory infiltrate was present in the interlobar and peri-portal spaces. However, these alterations in the livers of PCOS rats fed ISF-0·5 or ISF-1 were more discrete, with some points of inflammatory infiltration in the central lobular vein and a discrete quantity of vacuoles in the hepatocytes (Fig. 3(a)-E and -F). Therefore, soya ISF showed a moderate hepato-protective role against the negative response in PCOS.

The DHT-treated rats had significantly larger areas of lipid droplets accumulated in the livers (Fig. 3(a)-D) compared with the rats without DHT treatment (non-PCOS; Fig. 3(a)-A, -B and -C). The intake of soya ISF dose-dependently reduced DHT-induced accumulations of lipid droplets in the livers (Fig. 3(a)-E and -F). These effects were confirmed using oil red O staining (Fig. 3(b)) and quantification of the areas of lipid droplets in the livers (Fig. 3(c), p < 0·0001).

### Serum and hepatic lipid content

The rats fed 1 g/kg diet of soya ISF but not treated with DHT had lower serum TC, FC, NEFA and leptin levels compared with the Control (< 0·05). The PCOS rats had higher serum TC, FC, TAG, NEFA and leptin and lower LDL-cholesterol. Dietary soya ISF at both doses reduced serum NEFA and elevated serum TAG levels in the rats treated with DHT. Soya ISF at 1 g/kg diet markedly reduced hepatic TAG content in the rats with no DHT treatment and lowered serum leptin levels in the PCOS rats compared with their respective Controls (*P* < 0·05). PCOS rats exhibited increased hepatic TC and NEFA content compared with the non-PCOS Control (Table 1, *P* < 0·05).

**Table 1.** Serum and hepatic lipid levels of the rats treated with 0 µg/d (Control) or 83 µg/d dihydrotestosterone (DHT) and fed different levels of soya isoflavones (ISF) for 8 weeks (Mean values with their standard errors)

		Control						DHT								
		ISF-0		ISF-0·5		ISF-1		ISF-0		ISF-0·5		ISF-1		*P*		
	Lipids	Means	sem	Means	sem	Means	sem	Means	sem	Means	sem	Means	sem	DHT	ISF	DHT × ISF
Serum*	(mg/ml)															
	Total cholesterol	1·25	0·05a	1·21	0·06ab	1·068	0·07b	1·36	0·05	1·31	0·07	1·22	0·06	0·0179	0·0396	NS
	Free cholesterol	0·19	0·01a	0·20	0·01a	0·16	0·01b	0·22	0·01	0·22	0·01	0·21	0·01	0·0005	0·0486	NS
	LDL-cholesterol	0·59	0·05	0·61	0·04	0·59	0·05	0·42	0·05	0·38	0·03	0·44	0·04	< 0·0001	NS	NS
	HDL-cholesterol	0·12	0·02	0·09	0·01	0·10	0·01	0·08	0·01	0·08	0·01	0·10	0·02	NS	NS	NS
	TAG	0·61	0·07	0·64	0·06	0·67	0·09	1·22	0·12a	1·65	0·14b	1·52	0·16b	< 0·0001	NS	NS
	NEFA	0·47	0·03a	0·37	0·03ab	0·33	0·02b	0·51	0·03a	0·40	0·03b	0·43	0·03b	0·0226	0·0004	NS
	Leptin (ng/ml)	10·04	0·87a	9·10	0·77ab	4·90	0·47b	13·14	1·82a	11·06	1·51ab	8·96	0·76b	0·0018	0·0004	NS
Liver*	(mg/g liver)															
	Total cholesterol	1·69	0·16	1·79	0·09	1·73	0·05	2·21	0·21	2·02	0·15	2·18	0·16	0·0012	NS	NS
	TAG	12·11	1·33a	10·17	0·84ab	6·69	0·43b	14·05	1·92	12·00	1·37	11·60	1·20	0·0064	0·0103	NS
	NEFA	0·74	0·05	0·79	0·04	0·69	0·02	0·86	0·03	0·77	0·06	0·80	0·03	0·0345	NS	NS

* Values are means (sem), *n* 26 for serum lipids except for leptin (*n* 12) and eighteen for liver samples. Means with different letters within Control or DHT groups differ, *P* < 0·05. *P* > 0·05.

### Morphological and histological changes in ovaries

**Fig. 4.**
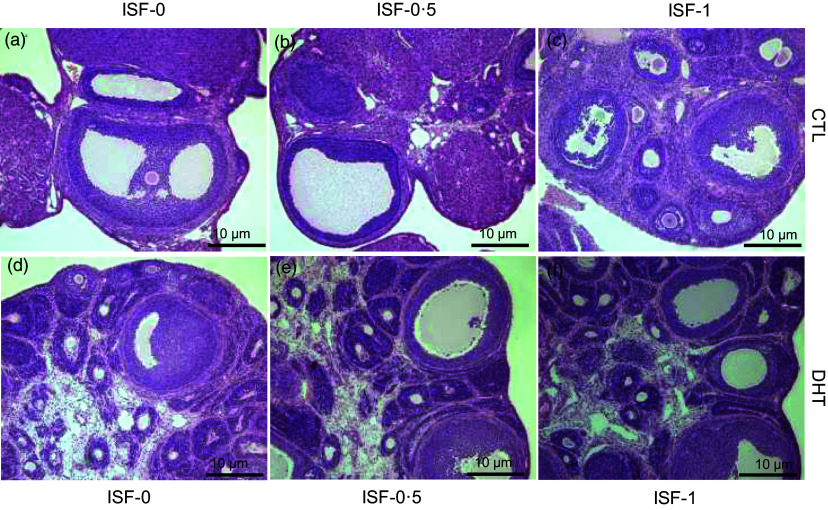
Ovary histomorphology with haematoxylin and eosin staining in the rats treated with 0 µg/d (Control, open bars) or 83 µg/d (DHT, solid bars) of dihydrotestosterone and fed diets containing either 0, 0·5, or 1 g/kg diet of soya isoflavones for 8 weeks. The images shown are representatives of ten replicates of each treatment group. *n* 3. ISF, isoflavones.

The ovaries in non-PCOS rats (no DHT treatment), regardless of their dietary ISF intake, were covered with simple or cuboidal epithelium and had numerous follicles, luteal bodies and interstitial cells within them (Fig. 4(a), (b) and (c)). The interstitial cells were polyhedral cells with central and spherical nuclei situated between the ovarian follicles. There were numerous ovarian follicles at different stages of development, including primordial, unilamellar, multilamellar, antral and mature, and many corpora lutea in various stages of functioning were observed.

In all DHT-treated groups, the ovaries were atrophied with numerous cystic follicles, other leukocyte infiltrates and the absence of luteal bodies (Fig. 4(d)). In the ovarian follicles, the presence of an internal theca layer forming spherical structures or epithelioid-like cords with pyknotic nuclei (indications of apoptosis) is observed, along with interstitial cords. Considerable ovarian atrophy was also observed in the DHT-treated rats fed with 0·5 and 1 g/kg ISF (Fig. 4(e) and (f)). Therefore, anovulation was histologically evident in the DHT groups and confirmed by the persistent diestrus, indicating a failure of the ovaries to release the oocyte.

### Regularity of oestrous cycles and distribution of ovarian follicles

**Fig. 5.**
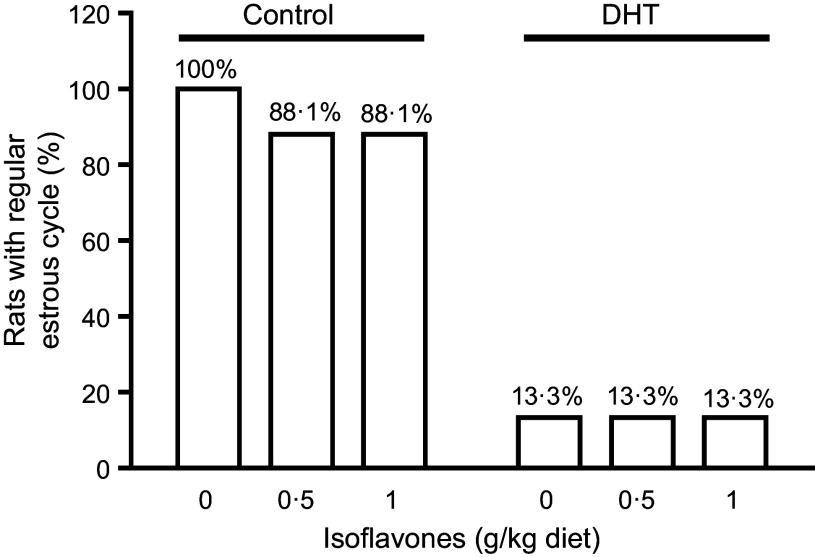
Percentages of the rats with regular oestrous cycle measured in the last 2 weeks prior to the end of the 8 weeks’ treatment with 0 µg/d (Control, open bars) or 83 µg/d (DHT, solid bars) of dihydrotestosterone and fed diets containing either 0, 0·5 or 1 g/kg diet of soya isoflavones. *n* 8.

Most of the PCOS rats did not exhibit regular oestrous cycles and remained in the diestrus phase (86·7 %, Fig. 5). DHT treatment significantly increased the percentage of primordial follicles (60 % *v*. 50·9 %) and decreased the percentage of primary follicles (13·8 % *v*. 30·2 %) in the ovaries compared with the Control (Table 2, *P* < 0·05). Soya ISF at both levels reduced the primordial follicles (45·6 % for ISF-0·5 and 51·3 % for ISF-1 *v*. 60 % for ISF-0) and increased the primary follicles (34·2 % for ISF-0·5 and 27·6 % for ISF-1 *v*. 13·8 % for ISF-0) in the ovaries of the rats treated with DHT compared with the Sham Control (Table 2, p < 0·05).

**Table 2.** Follicle populations in the ovaries of the rats treated with 0 µg/d (Control) or 83 µg/d dihydrotestosterone (DHT) and fed different levels of soya isoflavones (ISF) for 8 weeks (Numbers and percentages)

	Control						DHT					
	ISF-0		ISF-0·5		ISF-1		ISF-0		ISF-0·5		ISF-1	
Follicles	*n*	%	*n*	%	*n*	%	*n*	%	*n*	%	*n*	%
Primordial	81	50·9*a	66	48·2a	57	47·9a	117	60b	72	45·6a	78	51·3a
Primary	48	30·2a	42	30·7a	31	26·1a	27	13·8b	54	34·2a	42	27·6a
Secondary	9	5·7	10	7·3	11	9·2	19	9·7	14	8·9	16	10·5
Graafian	21	13·2	19	13·9	20	16·8	32	16·4	18	11·4	16	10·5
Total	159	100	137	100	119	100	195	100	158	100	152	100

* Follicle scores (%), the scores in the same row with different letters differ, *P* < 0·05, *n* 3.

## Discussion

The results of present studies have shown that treatment with DHT, a metabolite of testosterone, significantly increased food intake, BWG, accumulation of lipid droplets in the liver (a key parameter of NAFLD) and insulin resistance in the female Sprague-Dawley rats. Furthermore, DHT markedly reduced ovary weight, length, and percentage of primary follicles, while increased the percentage of primordial follicles in the ovaries compared with the Control. Most of the rats treated with DHT had irregular oestrous cycle and stayed at the diestrus phase. Dietary ISF supplementation at both levels tested in the present study attenuated DHT-induced increase in BWG, hepatic accumulation of lipid droplets and suppression in ovarian follicular development. DHT-induced insulin resistance was mitigated by a higher level of ISF intake (1 g/kg diet).

The present study showed that at a higher dose of 1 g/kg diet, ISF alone reduced BWG without affecting food intake, suggesting that ISF may reduce the bioavailability of nutrients. This is consistent with the observation that soya ISF reduced weight gain without affecting food and energetic intake in ovariectomized rats fed a high-fat diet^(29)^ and significantly reduced digestibility of nutrients including dry matter, crude protein, crude fibre and diethyl ether extract^(30)^. DHT treatment increased both BWG and food intake in the present study. Similar effects have also been reported in women with PCOS^(31)^ and rats treated with DHT^(22)^. In ovariectomized mice with free access to food, DHT treatment increased food intake, body weight and fat accumulation in the liver and impaired glucose tolerance and leptin sensitivity. However, when these mice were fed a restricted diet and had the same energetic intake as the control animals, their BWG was not different from that of the control animals, suggesting that the DHT-induced weight gain was due to increased food intake possibly mediated through altered leptin sensitivity^(31)^. Leptin is produced by adipocytes and plays a crucial role in regulating food intake, body weight and energy balance^(32)^. Our study has shown that DHT elevated serum leptin concentrations compared with the Control group. Dietary ISF at a higher dose (1 g/kg diet) significantly reduced serum leptin levels in both the DHT-treated and untreated rats compared with their respective Control groups. The precise roles played by DHT-induced leptin in the development of PCOS in the present study remain to be determined. Similar findings have been reported in both patients with PCOS^(33)^ and DHT-treated rats^(22)^. However, the contribution of leptin to the development of other metabolic disorders in PCOS appears inconsistent. While circulating leptin levels showed positive correlations with BMI, insulin and androgen levels in women with PCOS and obesity^(34–36)^, this relationship seems to be not significant in the lean patients with PCOS^(37–40)^.

On the other hand, oestrogen also plays a crucial role in regulating energy balance, food intake and body fat distribution in females. Decreased oestrogen levels can result in obesity in rodents and humans^(41)^. Ovariectomy, which causes a decrease in oestrogen levels, has been shown to increase body weight and fat accumulation in the liver and perirenal area in mice. However, treatment with ISF-enriched soyabean leaves inhibited ovariectomy-induced weight gain and fat accumulation. The underlying mechanism involved in the effects of ISF might be due to their oestrogenic actions and roles in the restoration of the decreased ERβ and ER-mediated PI3K/Akt signalling pathway in the hippocampus^(42)^. The ER-mediated PI3K/Akt signalling pathway plays important roles in regulating energy homeostasis through balancing energy expenditure and energy intake^(43)^. Activation of this pathway by the administration of oestrogen or oestrogenic compounds leads to increased mitochondrial function and energy expenditure. Meanwhile, it also attenuates the ovariectomy-induced increase in neuropeptide Y, thereby reducing the central orexigenic or appetite-stimulating action and causing decreased food intake and weight gain^(44,45)^.

DHT increased blood glucose levels and caused insulin resistance in the present study. A higher level of ISF (1 g/kg diet) reduced DHT-induced insulin resistance, suggesting that ISF may have preventive or therapeutic benefits in mitigating androgen-induced glucose imbalances. This is consistent with the results from studies in humans and rats. The intake of 50 mg/d soya ISF containing 37·5 mg genistein, 10 mg daidzein and 2·5 mg glycitein for 12 weeks in women with PCOS improved markers of insulin resistance, such as reduced serum insulin and insulin resistance estimated using homeostasis model assessment and increased quantitative insulin sensitivity check index^(18)^. In rats, DHT caused insulin resistance^(22)^, while ISF improved insulin sensitivity and reduced serum insulin levels^(29)^. Administration of genistein alleviated insulin resistance and improved hormone balance in rats with PCOS induced by oestradiol valerate^(25,46)^. Furthermore, genistein attenuated the increase in the fasting blood insulin level and homeostasis model assessment in letrozole-induced PCOS rats^(47,48)^. Equol is one of the most active ISF metabolites and acts as a selective ER modulator. In patients with PCOS who underwent a defined ISF intervention using soya milk, higher equol production was linked to lower androgen and fertility markers. The glucose homeostasis in patients with PCOS was improved to a level similar to that of the Control group at baseline measurements^(49)^. Overall, these studies suggest that soya ISF or genistein may have preventive or therapeutic potential for improving insulin sensitivity and reducing the adverse metabolic effects of androgen in patients with PCOS and animal models of PCOS.

We have also found that DHT increased lipid droplet accumulation, TC, TAG and NEFA in the liver. Additionally, DHT elevated serum TC, FC, TAG and NEFA while reducing LDL-cholesterol. ISF reduced DHT-induced hepatic accumulation of lipid droplets and reduced serum TC, FC and NEFA while increasing TAG levels. ISF supplementation showed a protective role against the adverse effects of DHT on the liver and serum, and these results corroborate with others using the neonatal female rats treated with testosterone. In these rats, testosterone caused histological changes in the liver that mimic NAFLD, impaired metabolism of branched-chain amino acids and dysfunctions in the activity of liver fatty acid elongase-2^(50)^. In the oestradiol valerate-induced PCOS rats, ISF administration reduced serum TAG and cholesterol levels and improved HDL levels. Serum LDL levels were reduced in rats fed higher dosages of ISF (150 and 200 mg/kg) after 3 months^(30)^. In women with obesity, hyperinsulinemia and dyslipidemia, who also have PCOS, genistein supplementation improved TC levels and reduced LDL-cholesterol and the LDL:HDL ratio, while TAG showed a trend towards a decrease^(16)^. Overall, these findings suggest that DHT adversely affects lipid metabolism in the liver, while ISF or genistein may have some protective effects.

In the present study, we have shown that DHT reduced ovary weight and length, disrupted the regularity of oestrous cycle and caused ovarian atrophy and suppression of follicular development. Soya ISF alleviated the suppressive effect of DHT on ovarian follicular development from primordial to primary follicles but failed to rescue the histological feature of the ovaries and the irregularity of oestrous cycles in the DHT-treated rats. In the letrozole-induced PCOS rats, administration of soya ISF after PCOS induction decreased the percentage of the diestrus phase and resulted in well-developed antral follicles and a normal granulosa cell layer in the ovary^(48)^ and could also reduce the severity of menstrual irregularity and polycystic ovaries^(51)^. Genistein administration increased luteinisation and reduced cystic follicles in the same rat model^(47)^. These beneficial effects of soya ISF in the letrozole-induced PCOS rat model are believed to be due to their ability to reduce testosterone concentration in the peripheral blood through the inhibition of letrozole-induced increase in steroidogenic enzyme activity, including 3β-hydroxy steroid dehydrogenase and 17β-hydroxy steroid dehydrogenase^(48)^. However, in our study, DHT had been administered at a constant level that was not altered by the supplemented soya ISF. This might be the reason soya ISF had no significant effects on DHT-induced changes in the ovarian weight, length morphology and the irregularity of oestrous cycles.

In summary, our results have shown that dietary supplementation with soya ISF mitigated DHT-induced BWG, insulin resistance and lipid droplet accumulation in the livers of a PCOS rat model. This suggests that the consumption of soya foods or ISF supplements may be beneficial for individuals with PCOS in alleviating associated metabolic disorders, such as diabetes and NAFLD. However, soya ISF failed to restore the histomorphological features of the ovaries and reverse the irregularity of the oestrous cycle in DHT-treated rats. This might be a limitation of the DHT-induced PCOS rat model, in which a constant dosage of exogenous DHT used to induce PCOS counteracts the actions of soya ISF in the modulation of endogenous testosterone production. However, in models inducing high levels of endogenous testosterone, such as the letrozole-induced PCOS model, soya ISF or genistein could modulate the production of endogenous sex hormones, thereby restoring the ovarian functions and regularity of oestrous cycles.

## Supporting information

Xiao et al. supplementary material Xiao et al. supplementary material

## Supplementary material

For supplementary material/s referred to in this article, please visit https://doi.org/10.1017/S0007114524001296.
