# Supplementary figures and images for: Consumption of soya isoflavones improved polycystic ovary syndrome-associated metabolic disorders in a rat model
Source: Br J Nutr. 2024 Jun 3;132(4):416–24. doi: 10.1017/S0007114524001296 (PMC11499085; doi:10.1017/S0007114524001296)

## Experimental Design

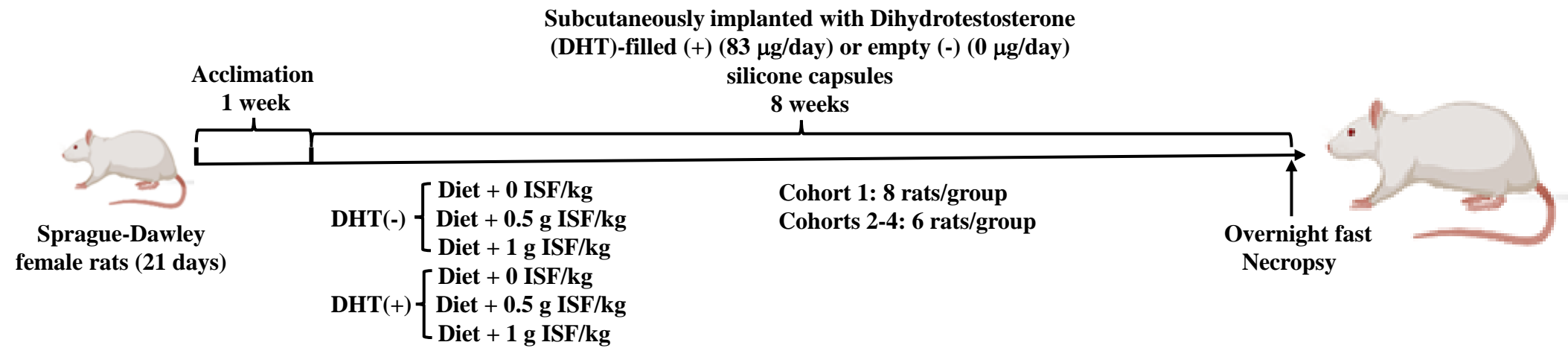

Supplementary Figure-1

Supplement: Xiao et al. supplementary material [file S0007114524001296sup001.pdf]
